# COVID-19-Related Mental Health Effects in the Workplace: A Narrative Review

**DOI:** 10.3390/ijerph17217857

**Published:** 2020-10-27

**Authors:** Gabriele Giorgi, Luigi Isaia Lecca, Federico Alessio, Georgia Libera Finstad, Giorgia Bondanini, Lucrezia Ginevra Lulli, Giulio Arcangeli, Nicola Mucci

**Affiliations:** 1 Department of Human Sciences, European University of Rome, via degli Aldobrandeschi, 190, 00163 Rome, Italy; gabriele.giorgi@unier.it; 2 Department of Experimental and Clinical Medicine, University of Florence, Largo Brambilla, 3, 50134 Florence, Italy; luigiisaia.lecca@unifi.it (L.I.L.); nicola.mucci@unifi.it (N.M.); 3 Business @ Health Laboratory, European University of Rome, via degli Aldobrandeschi, 190, 00163 Rome, Italy; federico.alessio94@gmail.com (F.A.); g.liberafinstad@gmail.com (G.L.F.); giorgia.bondanini@gmail.com (G.B.); 4 School of Occupational Medicine, University of Florence, Largo Brambilla, 3, 50134 Florence, Italy; lucreziaginevra.lulli@unifi.it

**Keywords:** SARS-CoV-2, COVID-19 pandemic, occupational health and safety, mental health, psychological disorders, workplace organization

## Abstract

The Coronavirus Disease 2019 (COVID-19) pandemic has deeply altered social and working environments in several ways. Social distancing policies, mandatory lockdowns, isolation periods, and anxiety of getting sick, along with the suspension of productive activity, loss of income, and fear of the future, jointly influence the mental health of citizens and workers. Workplace aspects can play a crucial role on moderating or worsening mental health of people facing this pandemic scenario. The purpose of this literature review is to deepen the psychological aspects linked to workplace factors, following the epidemic rise of COVID-19, in order to address upcoming psychological critical issues in the workplaces. We performed a literature search using Google Scholar, PubMed, and Scopus, selecting papers focusing on workers’ psychological problems that can be related to the workplace during the pandemic. Thirty-five articles were included. Mental issues related to the health emergency, such as anxiety, depression, post-traumatic stress disorder (PTSD), and sleep disorders are more likely to affect healthcare workers, especially those on the frontline, migrant workers, and workers in contact with the public. Job insecurity, long periods of isolation, and uncertainty of the future worsen the psychological condition, especially in younger people and in those with a higher educational background. Multiple organizational and work-related interventions can mitigate this scenario, such as the improvement of workplace infrastructures, the adoption of correct and shared anti-contagion measures, including regular personal protective equipment (PPE) supply, and the implementation of resilience training programs. This review sets the basis for a better understanding of the psychological conditions of workers during the pandemic, integrating individual and social perspectives, and providing insight into possible individual, social, and occupational approaches to this “psychological pandemic”.

## 1. Introduction

In late December 2019, a number of local health authorities of Wuhan, Hubei Province in China, reported clusters of patients with pneumonia of an unknown cause, which were epidemiologically linked to a seafood market in Wuhan [1]. The first case was reported by the World Health Organization (WHO) on 31 December 2019. However, some experts believe that the earliest case of COVID-19 was detected as early as 17 November 2019 [2]. The pathogen, a novel coronavirus (SARS-CoV-2), was identified by local hospitals, as stated by the WHO on 9 January 2020. Subsequently, COVID-19 has spread rapidly throughout the world and has reached pandemic proportions affecting all continents. The WHO declared the outbreak a public health emergency of international concern on 30 January 2020, when all 34 regions of China showed cases of infection and the total number of infections exceeded that of severe acute respiratory syndrome (SARS) of 2003. On 11 March 2020, the outbreak was declared a global pandemic [3]. By 26 March 1.7 billion people worldwide were under some form of lockdown, which increased to 3.9 billion people by the first week of April, in other words, more than half of the world’s population

From the beginning of the pandemic outbreak to date (23 July 2020), the following data emerge from the COVID-19 online dashboard of the Center for Systems Science and Engineering (CSSE) of the Johns Hopkins University (JHU): 15,239,805 actual and confirmed cases worldwide, 623,507 global deaths, 8,656,734 global recovered, and a total of 188 countries and territories with at least one COVID-19 case.

The 2019 coronavirus epidemic can undermine not only physical health but also individuals’ psychological resources and resilience. In a highly interconnected and globalized world, the impacts of the pandemic on a social and economic level have become evident since the outbreak [4]. The global economy has slowed down sharply and global stock indices have plunged [4]. A lot of people committed suicide [5,6], and millions of people lost their jobs [7]. The press release of the International Labor Organization (ILO) of 18 March 2020, reported a drop of 24.7 million jobs as the worst-case scenario and 5.3 million as the best scenario. In the worst-case scenario, the world unemployment rate would rise from 4.936% to 5.644%, along with an increase in suicides of around 9570 per year. In the worst case scenario, unemployment would rise to 5.088% along with an increase of approximately 2135 suicides [8]. Moreover, the economic and productive consequences of the pandemic can affect job sectors differently. While some workers were substantially involved in countering the rise of COVID-19, others were forced to stop their work activity due to lockdown policies or effective job loss. Where possible, some companies have experienced a high increase of new organizational methods, such as smart working.

The pandemic could have severe effects on the mental health of the general population and of workers. Experts point out that both people who already suffered from psychiatric problems, and others who have never experienced symptoms, could be at risk [9].

In this pandemic scenario, some work-related and organizational factors could play a crucial role in exacerbating or moderating the effect on people’s mental health. Therefore, in addition to the medical or economic implications, it is essential to analyze the psychological side of the pandemic and the factors related to mental health in the workplace. The various psychological problems that will arise once the acute coronavirus emergency phase has passed are not receiving the necessary attention. In this way, there is a risk of witnessing the presence of another “pandemic” around the world linked to the development of possible mental disorders. In a recent study, Gunnel and colleagues [10] provided accurate predictions on how the effects on mental health of the pandemic could, in turn, have an important psychological impact on the whole population. Therefore, research data for the development of evidence-based approaches are essential to reduce the negative consequences of the epidemic on psychological health [10].

### 1.1. Theoretical Background

It is well established that the 2019 coronavirus pandemic could have an important psychological impact [9]. Due to the deep changes determined by the SARS-CoV-2 in the workplaces, and in the way to perform work activities, it can be hypothesized that some occupational and organizational factors could play a relevant role in the mental health of workers and their ability to cope with a new challenging working scenario. It has been widely demonstrated that the work environment, work organization, and work-related behaviors are factors capable of influencing mental health and psychological well-being of workers [11]. It is plausible that those factors could be influenced by the pandemic, contributing to exacerbate or moderate mental health outcomes. In fact, numerous stressors that employees face in a pandemic can affect different aspects of the workplace.

Being that COVID-19 is a communicable disease, some factors related to the risk of contagion in the workplace and the adoption of preventive procedures can cause several mental concerns. For example, the lack personal protective equipment (PPE), the physical weight caused by wearing them, the fear of being infected and that this could harm family members, the conflict between safety procedures and the desire to provide support, longer working hours, pressing multitasking and the stigmatization of people working in high-risk environments can deeply affect mental well-being of workers. In response, workers may develop a range of behavioral (e.g., consequences on performance), physical (e.g., headache, gastric disturbances), and psychological (e.g., mood swings, lowered motivation, depressive thoughts, and isolation) reactions [12].

Although the pandemic constitutes a universal hazard for all professional categories, it is possible to trace high-risk populations (e.g., healthcare personnel). During acute health crises, the healthcare sector is subjected to an excessive strain that adversely affects working life [13]. In a pandemic, the number of patients increases significantly, placing additional stress on staff and undermining healthcare resources. Furthermore, doctors perceive a greater risk for themselves due to their exposure to patients—adding further stress [14,15]. Lai et al. [16] examined the mental health status of 1257 doctors and other healthcare professionals in China. 50.4% of study participants reported depression, 44.6% anxiety, 34.0% insomnia, and 71.5% distress. This stressful situation is further complicated by the shortage of personal protective equipment (PPE) that can arise during a pandemic [17]. The perceived risk of being infected is justified: a meta-analysis of the professional risk resulting from the 2009 swine flu pandemic (influenza A H1N1) showed that the chances of healthcare professionals contracting the virus were double that of the control groups [18]. This increased risk may be due to greater exposure to patients’ respiratory secretions [19]. Another stressor is the increased risk of contagion for families of frontline healthcare workers [20]. Swine flu pandemic data from 2009 show that 20% of healthcare workers with symptoms reported symptoms in at least one of their family members [21]. One way for frontline healthcare providers to decrease the risk of infection for their families is through social distancing. Nevertheless, the role of social support in moderating the stress response is well demonstrated [22] and social distancing deprives the subject of a crucial defense against negative effects on psychological health precisely in the moment of greatest suffering [23].

Furthermore, one of the collateral phenomena of the COVID-19 pandemic is the progressive stigma that is spreading alarmingly, as evidenced by a large body of research [24,25]. The categories most exposed to discrimination and stigma are infected people and healthcare workers. The stigma towards COVID-19 patients increases the risk of psychopathology (e.g., depressive symptoms, stress-related disorders, and sleep disorders). Those who have been quarantined may also have problems returning to work. This delicate aspect highlights how work implications are extremely important for the well-being of the individual. Experiencing stigma and discrimination in the workplace could also lead to loss of productivity and income [26]. The results of a study on the effects of SARS epidemic showed how people who had healed experienced the stigma of family members, peers and co-workers [27]. Indeed, a further pivotal aspect concerns the inability to access employment and to resume one’s work, with devastating consequences for the individual [28]. On the other hand, healthcare workers represents the professional category that suffers most from the consequences of stigma [29,30,31]. As a result, there is an increased risk of burnout, psychological distress, emotional exhaustion, anxiety and depressive symptoms [16,26,32]. Not being socially supported due to stigma could also affect workers’ self-efficacy level [33].

The recent research by Ramaci et al. carried out on a sample of 260 healthcare workers from an Italian hospital, analyzed the impact of stigma on work outcomes [34]. The results of the study show how stigma positively predicts burnout and fatigue and negatively predicts satisfaction, highlighting the importance of discriminatory behavior. In this perspective, the application of human resources (HR) practices to decrease the weight of discrimination becomes crucial [34].

Social exclusion is also negatively associated with mental health of migrants [35]. Internal migrant workers experience high levels of anxiety, psychotic, and post-traumatic disorders due to adverse socio-environmental conditions, such as loss of social status and discrimination [36]. In addition to the problems created by the pandemic, public health strategies, such as mandatory isolation, or quarantine in governments’ temporary shelters, or the call for people to return to their original places, and social distancing, increase the feeling of loneliness, leading to mental problems that can contribute to suicide.

Based on what has been described, the current situation calls for the use of evidence-based best practices capable of moderating the negative effects of the pandemic on workers’ mental health.

### 1.2. Aim of the Narrative Review

The management of work-related factors affecting mental health in a pandemic scenario seems crucial to support people engagement and consequently psychological well-being. This is of special interest to those professionals directly involved in the COVID-19 contrast actions, but also to the overall workforce dealing with new organizational approaches, different ways of working and other work related factors such as returning to work after a period of interruption, job loss, job insecurity, and fear of the future due to a possible business failure. For these reasons, there is a need to provide evidence on how organizational and work-related factors can contribute to maintain or affect psychological well-being.

The purpose of the following narrative review is to provide a general overview of the various psychological and social implications linked to work related factors, following the current SARS-CoV-2 pandemic. In particular, this narrative review aimed to describe and acknowledge how psychological aspects resulting from the outbreak of the SARS-CoV-2 epidemic could be linked to various workplace and organizational factors, in order to help researchers and stakeholders to entail targeted strategies aimed at managing psychological health outcomes related to the occupational scenario.

## 2. Materials and Methods

The literature search was performed during July 2020 using Google scholar, PubMed, and Scopus as databases. As inclusion criteria, we considered only articles in the English language, and only studies performed in humans. As publication type, we considered articles in scientific journals, letters to editor, comments, and book chapters. We restricted the literature search for articles published in the last year (December 2019–July 2020), while the historical background has been written without time restrictions. Following the population, intervention, comparison, outcomes (PICO) strategy for scientific research [37], we used a specific string of search. In order to include relevant literature about the theme, we combined several search terms belonging to each PICO section:

- Population: workers, employees;
- Intervention: workplace, organization, job, job task, occupation, occupational;
- Comparison: COVID-19, SARS-CoV-2, 2019-nCoV, coronavirus, epidemic, pandemic;
- Outcome: mental health, mental illness, psychological health, stigma, psychological disorders, stress, post-traumatic stress disorder, depression, suicide.

**Figure 1:**
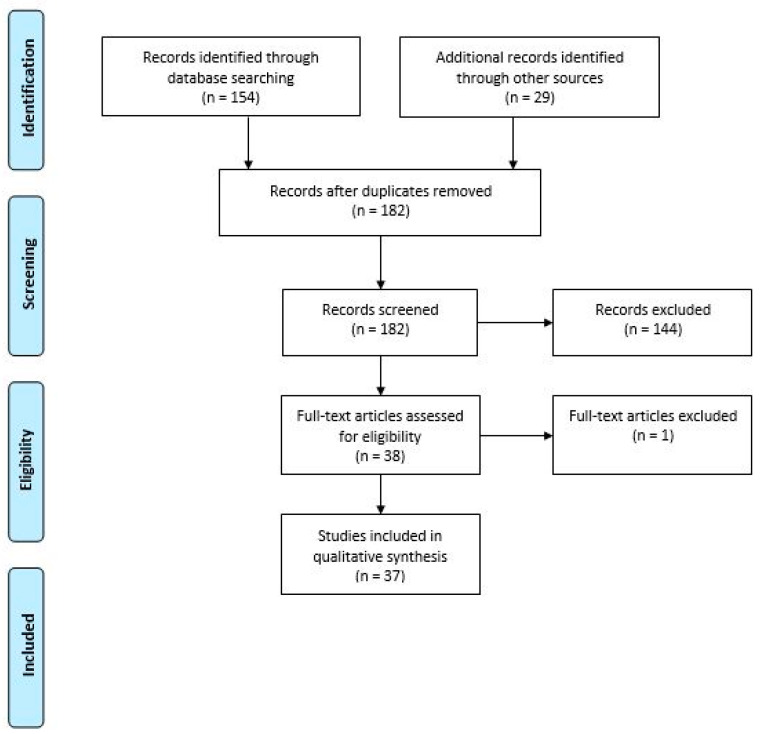
Flow diagram of the literature search strategy and review process, following PRISMA 2009 flow diagram rules.

A total of 183 articles were collected and screened using a title-abstract analysis. All of the studies that did not consider occupational or organizational factors in the relationship between COVID-19 pandemic and mental health were excluded. Only articles that related to organizational and work-related factors on the psychological and mental health consequences of COVID-19 were then included and considered for a full-text content analysis. The judgement about the inclusion of each paper was performed separately by the investigators L.I.L. and F.A. In case of disagreement, the decision was made collegially with the contribution of a third investigator, G.G. Figure 1 shows a flow-diagram of the literature search strategy and the review process following PRISMA 2009 flow diagram rules [38].

After the exclusion of 145 non-relevant articles, we included 37 full-text articles to critically evaluate the workplace related factors that demonstrated an influence on psychological and mental health during the COVID-19 pandemic.

## 3. Results

Thirty-seven articles that met inclusion criteria in the title-abstract reading stage were identified and evaluated. The summary of the included articles is reported in Table 1.

The included studies found several occupational factors as being able to influence workers’ mental health outcomes in the COVID-19 pandemic scenario.

Several studies considered job task as a risk factors for the onset of mental related issues. In particular, the majority of the studies considered healthcare workers and frontline workers as a work group at higher risk of developing several psychological outcomes such as depression, anxiety, stress, sleep disturbance and so on. Evidence demonstrates that COVID-19 pandemic caused sleep disturbances and suicidal thoughts in healthcare workers [39]. The SARS-CoV-2 epidemic brings high levels of psychological distress, insomnia, alcohol, and drug misuse, and symptoms of post-traumatic stress disorder (PTSD), depression, and higher perceived stress primarily on younger people, medical staff and all healthcare and emergency workers, which seems to be the most affected categories [40,41,42]. In a similar way, Horsch et al., (2020) clarified how SARS-CoV-2 epidemic will inevitably lead to depression, anxiety, and work-related problems for healthcare workers [43].

A relevant body of studies (number: 21) considered the impact of organizational factors on moderating or exacerbating the effect of COVID-19 on mental health. In particular, on the one hand, work related stress seems to exacerbate mental health issues, as well as poor social support and a prolonged working time. On the other hand, the availability of secure procedure to manage the risk of contagion and the availability of personal protective equipment seems to moderate the risk of mental health concerns. Concerning suicide cases, the results of the qualitative analysis enlighten underlying reasons, such as fear of COVID-19 infection, financial crisis, loneliness, social boycott, pressure for quarantine, fear of positive COVID-19, and pre- and post- lockdown work-related stress [44]. Some common and social measures, such as quarantine and delays in returning to work, were also associated with mental health [45]. In addiction, psychological help has been considered very useful although administered via social media [46]. Cognitive behavioral therapy (CBT), motivational interviewing (MI), and/or crisis intervention have been considered useful intervention strategy for the management of mental health outcomes in healthcare workers. Huang and Zhao (2020) observed higher levels of stress related to how often people think about the epidemic [40]. Thus, the return to work appeared as a relevant factor to stop ruminant thoughts on the pandemic.

Reducing working time, enhancing smart working, promoting secure protocols, trainings, and improving job/leadership support seems to be related to better performance and well-being. Above all, security and safety equipment seem to be highly and positive related to workers well-being and performance (6/42). The study of Sasaki et al. [47] showed how the amount of prevention measures was negatively associated with the psychological distress of the employees and positively associated with their performance, suggesting how rigorous prevention measures reduce psychological distress, protecting work outcomes.

Some studies considered the impact of COVID-19 pandemic on mental health outcomes in vulnerable working populations. The most vulnerable workers categories seems to be the front-line workers and health care workers, migrants, and young adult workers. In particular, results by Choudhari (2020) suggest that the professional community of internal migrant workers is prone to the development of psychological effects due to the disturbing double impact of the COVID-19 crisis and the related adverse professional scenario [48]. Similar results were obtained by the study of Chander et al. in a cohort of Indian migrant workers [49].

**Table 1:** Summary of included articles.

Title	Authors	Type of Study/Methods	Sample (If Available)	Study Setting and Main Results	Occupational Factor Considered	Outcomes
Nurses’ Mental Health and Well-Being: COVID-19 Impacts [50].	Stelnicki AM, Carleton RN, Reichert C.	Narrative review/literature search	N.A.	This study examined the mental health of healthcare workers, such as nurses after the peak of the COVID-19 pandemic. Large-scale disasters have been accompanied by an increase in symptoms of depression, post-traumatic disorder, insomnia, and substance use, particularly in front-line workers.	Job task: health care workers	Negative psychological outcome
At the height of the storm: Healthcare staff’s health conditions and job satisfaction and their associated predictors during the epidemic peak of COVID-19 [51]	Zhang SX, Liu J, Afshar Jahanshahi A, Nawaser K, Yousefi A, Li J, Sun S.	Cross sectional/survey	304	This study reports the levels of mental health, anxiety, depression, distress, and job satisfaction of doctors, nurses, and healthcare staff (Sample of 304 HCP) in Iran during the highest number of total active COVID-19 cases. Results indicate that a substantial portion of the sample reached the cutoff levels of disorders in anxiety (28.0%), depression (30.6%), and distress (20.1%).	Job task: health care workers	Negative psychological outcome
Workplace responses to COVID-19 associated with mental health and work performance of employees in Japan [47]	Sasaki N, Kuroda R, Tsuno K, Kawakami N.	Cross sectional/online survey	1448	This study investigated the links between workplace measures implemented in response to COVID-19 with mental health and work performance of employees (sample n = 1448) in Japan. The preventive measures were assessed on an original scale (based on the conceptual categories of recommendation for workplace measures). Workplace measures correlated positively with respondents’ fear of and worry associated with COVID-19, negatively with psychological distress, and positively with work performance.	Job task: front-line workers; workplace outcome: job performance	Positive psychological Outcome
The mental health of doctors during the COVID-19 pandemic [52]	Galbraith N, Boyda D, McFeeters D, Hassan T.	Perspective piece/literature search	N.A.	The coronavirus disease 2019 (COVID-19) crisis places additional pressure on healthcare staff and on the healthcare system in general. This research underlines how such pressure brings a greater risk of psychological distress and high levels of work stress for doctors, nurses, and medical staff. Healthcare professionals place high value on provision of training and equipment during such pandemics, effective leadership, and managerial support for clinicians, and their families are also highly protective against negative psychological outcomes.	Job task: healthcare workers Organizational factors: Work related stress	Negative psychological outcome Positive psychological outcome
‘Policing’ a pandemic: Garda wellbeing and COVID-19 [53]	Rooney L, McNicholas F.	Perspective piece/literature search	N.A.	Studies investigating small-scale epidemics, such as Severe Acute Respiratory Syndrome (SARS), indicate that frontlines staff of an outbreak, when going to work every day, are exposed to an insuperable amount of stress and experience increased psychological morbidities as a result.	Job task: frontline staff Organizational factor: work commute	Negative psychological outcome
COVID 19 pandemic: Mental health challenges of internal migrant workers of India [48]	Choudhari R.	Narrative review/literature search	N.A.	One of the most vulnerable but neglected communities, the professional community of internal migrant workers, is prone to the development of psychological effects due to the disturbing double impact of the COVID-19 crisis and the related adverse professional scenario.	Workers population: migrant workers	Negative psychological outcome
Multidisciplinary research priorities for the COVID-19 pandemic: a call for action for mental health science [54]	Holmes EA, O’Connor RC, Perry VH, Tracey I, Wessely	Position paper	N.A.	This study concerns the psychological, social, and neuroscientific effects of COVID-19, and the establishment of immediate priorities and long-term strategies for mental health research. Mobilization will now allow us to apply the acquired learning to any future periods of major infection and lockdown, which will be particularly important for front-line workers and for vulnerable groups.	Job task: frontline workers. Workers population: vulnerable groups	Negative psychological outcome
Addressing the mental health concerns of migrant workers during the COVID-19 pandemic: An experiential account [49]	Chander R, Murugesan M, Ritish D, Damodharan D, Arunachalam V, Parthasarathy R, Raj A, Sharma MK, Manjunatha N, Bada Math S, Kumar CN.	Brief report	N.A.	Within India, a large proportion of people migrates (about 5000 migrant workers visited over 140 spots across the city of Bengaluru). The violent epidemic outbreak of SARS-CoV-2 has accentuated discrimination, work-rights exploitation, and job insecurity issues.	Vulnerable population: migrant workers	Negative psychological outcome
Is returning to work during the COVID-19 pandemic stressful? A study on immediate mental health status and psychoneuroimmunity prevention measures of Chinese workforce [55]	Tan W, Hao F, McIntyre RS, Jiang L, Jiang X, Zhang L, Zhao X, Zou Y, Hu Y, Luo X, Zhang Z, Lai A, Ho R, Tran B, HoC, Tam W.	Cross sectional/online survey	673	This study aims to quantify the immediate psychological effects and underlines psycho-neuroimmunity prevention measures of a workforce returning to work during the COVID-19 epidemic (sample: 673; mean age: 30.8; 74.4% male). Results indicate that about 3.8%, 3.7%, 1.5% and 2.3% of respondents reported moderate to severe anxiety, depression, stress, and clinical insomnia, respectively.	Organizational factors: return to work	Negative psychological outcome
Moral and mental health challenges faced by maternity staff during the COVID-19 pandemic [43]	Horsch A, Lalor J, Downe S.	Commentary	N.A.	The current COVID-19 pandemic places maternity staff at risk of engaging in clinical practice that may be in direct contravention with evidence. Research on previous epidemics and pandemics has shown the toll that patient care can have on the mental health of staff, such as elevated levels of psychological distress, insomnia, alcohol, and drug misuse, and symptoms of post-traumatic stress disorder (PTSD), depression, and higher perceived stress.	Job task: healthcare workers	Negative psychological outcome
Chinese mental health burden during the COVID-19 pandemic [40]	Huang Y, Zhao N.	Cross sectional/online survey	7236	The purpose of this study was to measure Chinese mental health during the COVID-19 pandemic. Data were collected from 7236 participants. Depressive symptoms, anxiety disorders, and poor sleep were assessed. Younger people and healthcare workers were at high risk for mental illness.	Job task: healthcare workers	Negative psychological outcome
Study on the public psychological states and its related factors during the outbreak of coronavirus disease 2019 (COVID-19) in some regions of China [42]	Wang Y, Di Y, Ye J, Wei W.	Cross sectional/online survey	600	The aim of this research is to show how the highly contagious power of SARS-CoV-2 will inevitably lead to depression, anxiety, and work problems for employees. A total of 600 questionnaire participants were psychologically stable. Non-anxiety and non-depression rates were 93.67% and 82.83%, respectively. There were anxiety in 6.33% and depression in 17.17%. Professionals, industrial service workers and other staff had a depression risk of 0.31 times and 0.38 times.	Job task: professionals, industrial service, other personnel	Negative psychological outcome
Aggregated COVID-19 suicide incidences in India: Fear of COVID-19 infection is the prominent causative factor [44]	Dsouza DD, Quadros S, Hyderabadwala ZJ, Mamun MA.	Cross sectional/search on local newspapers	69	This study presents 69 suicide cases due to the current pandemic. The reasons behind the suicide cases are fear of COVID-19 infection, financial crisis, loneliness, social boycott and pressure for quarantine, fear of positive COVID-19, pre- and post- lockdown, work related stress.	Organizational factors: work related stress	Negative psychological outcome
Prevalence of and Risk Factors Associated With Mental Health Symptoms Among the General Population in China During the Coronavirus Disease 2019 Pandemic [45]	Shi L, Lu ZA, Que JY, Huang XL, Liu L, Ran MS, Gong	Cross sectional/online survey	56,679	In China, through use of patient health questionnaires, the health of the population and symptoms of post-traumatic stress disorder, depression, anxiety, insomnia, and acute stress were assessed during the COVID-19 pandemic. Some measures, such as quarantine and delays in returning to work, were also associated with mental health.	Organizational factors: return to work	Mental health
COVID-19 pandemic: every day feels like a weekday to most [56]	Liu T, Meyerhoff J, Mohr DC, Ungar LH, Kording KP.	Cross sectional/online questionnaires	127	Psychological and behavioral changes during the early stages of the epidemic in the United States were examined in a longitudinal observational study, as there is a significant difference between mood and stress levels on weekdays and weekends and this implies a significant reduction of well-being of workers.	Organizational factors: working time	Stress levels and well-being
COVID-19: Presumed Infection Routes and Psychological Impact on Staff in Administrative and Logistics Departments in a Designated Hospital in Wuhan, China [57]	Luo LS, Jin YH, Cai L, Pan ZY, Zeng XT, Wang XH.	Case control/online questionnaires	18 cases, 18 controls	The purpose of this study is to explore the infection pathways and psychological impact of COVID-19 on staff (sample: 18) from administrative and logistic departments. A total of 88.89% thought have been infected by the working environment in hospitals, 77.78% of staff experienced psychological stress or emotional changes.	Job task: healthcare workers	Negative psychological outcome
COVID-19-Related Factors Associated with Sleep Disturbance and Suicidal Thoughts among the Taiwanese Public: A Facebook Survey [39]	Li DJ, Ko NY, Chen YL, Wang P, Chang YP, Yen CF, Lu WH.	Cross sectional/Online survey	1970	This study aims to analyze factors related to COVID-19 to understand how they are associated with sleep disturbances and suicidal thoughts among members of the public during the pandemic in Taiwan. Being a non-healthcare worker is a potential factor that could predict suicidal thoughts. Results also indicate that insufficient social support is a risk factor for depression, anxiety, and sleep problems among healthcare workers in the COVID-19 pandemic.	Job task: healthcare workers	Suicidal thoughts
Burnout syndrome in Romanian medical residents in time of the COVID-19 pandemic [58]	Dimitriu MCT, Pantea-Stoian A, Smaranda AC, Nica AA,	Narrative review/Literature search	N.A.	This article analyzed the relationship between burnout and activity of doctors in a non-COVID emergency hospital. The results indicate that young doctors (maximum 35 years) and doctors in non-COVID wards are more vulnerable. The existence of clear protocols, practical training, and protection measures reduces stress levels.	Job task: healthcare workers Organizational factors: practical training and protection measures	Negative psychological outcome
Mental health burden for the public affected by the COVID-19 outbreak in China: Who will be the high-risk group? [59]	Huang Y, Zhao N.	Cross sectional/online survey	7236	During the COVID-19 epidemic, it was noted that healthcare professionals were particularly at risk of experiencing psychological problems when they spent too much time thinking about the epidemic. In a sample of 7236 participants, the prevalence of anxiety symptoms and depressive symptoms was significantly higher in participants younger than 35 years. The prevalence of anxiety, depressive symptoms, and poor sleep quality was significantly higher in healthcare professionals. Authors proposed psychological aids.	Job task: healthcare workers	Negative psychological outcome
The relationship between COVID-19 knowledge levels and anxiety states of midwifery students during the outbreak: A cross-sectional web-based survey [60]	Sögüt S, Dolu İ, Cangöl E.	Cross sectional/online survey	972	The purpose of this study is to determine the relationship between the anxiety states in the workplace and knowledge levels of female midwifery students about COVID-19 during the outbreak. Results indicate that anxiety levels of the female students were high among those who visit the hospital during the pandemic and had parents or relatives who had chronic diseases. Female midwifery students had a high level of knowledge regarding COVID-19.	Job task: healthcare workers	Negative psychological outcome
Psychosocial burden of healthcare professionals in times of COVID-19—a survey conducted at the University Hospital Augsburg [61]	Zerbini G, Ebigbo A, Reicherts P, Kunz M, Messman H.	Cross sectional/questionnaires	111	The purpose of this study is to investigate the work and psychosocial burden of physicians and nurses based on their degree of contact with COVID-19 patients. Results indicate that nurses working in the COVID-19 wards reported higher levels of stress, exhaustion, and depressive mood, as well as lower levels of work-related fulfilment compared to their colleagues in the regular wards.	Job task: healthcare workers	Work-related stress and negative outcome
The psychological impact of COVID-19 pandemic on physicians in Saudi Arabia: a cross-sectional study [62]	Al Sulais E, Mosli M, AlAmeel T.	Cross sectional/online survey	529	The purpose of this study is to evaluate the impact that the SARS-CoV-2 pandemic has had on the workplace and on the psychological well-being of doctors. The study sample was 529 physician. Results indicate that the mostly common feelings reported by the participants during the pandemic were: worry (357, 67.5%), isolation (301, 56.9%), and fear (263, 49.7%).	Job task: healthcare workers	Negative psychological outcome
The Psychological Change Process of Frontline Nurses Caring for Patients with COVID-19 during Its Outbreak [63]	Zhang Y, Wei L, Li H, Pan Y, Wang J, Li Q, Wu Q, Wei H.	Cross sectional/interviews	23	The aim of this research is to identify the psychological change process of the registered nurses (n.23) who worked in the epicenter of the COVID-19 outbreak. The longitudinal study indicates the existence of three stages: early stage (ambivalence), middle stage (emotional exhaustion), and later stage (energy renewal). In addition, the recovery of patients or improvements of their conditions were also positive incentives for nurses.	Job task: healthcare workers	Negative psychological outcome
Generalized anxiety disorder, depressive symptoms and sleep quality during COVID-19 outbreak in China: a web-based cross-sectional survey [64]	Huang Y, Zhao N.	Cross sectional/online survey	7236	The purpose of this paper is to assess the mental health of Chinese workers during the epidemic and explore potential risk factors: healthcare workers and staff are at high risk for poor sleep quality.	Job task: healthcare workers	Negative psychological outcome
Psychological symptoms of ordinary Chinese citizens based on SCL-90 during the level I emergency response to COVID-19 [41]	Tian F, Li H, Tian S Yang J, Shao J, Tian C.	Cross sectional/online survey	1060	This study aims to analyze the psychological symptoms of citizens during the Level I emergency response across China. Analyzes revealed that healthcare workers are part of the high-risk group.	Job task: healthcare workers	Negative psychological outcome
An Integrative Total Worker Health Framework for Keeping Workers Safe and Healthy During the COVID-19 Pandemic [65]	Dennerlein JT, Burke L, Sabbath EL, Williams JAR, Peters SE, Wallace L, Karapanos M, Sorensen G.	Narrative review/literature search	N.A.	The purpose of this study is to promote an integrated Total Worker Health (TWH) approach, funded by the NIOSH, which includes human factors and ergonomic principles, supporting worker safety, health, and well- being during the COVID-19 pandemic. Results indicate that the approach can enhance human factors and ergonomics principles to improve well-being.	Organizational factors: human factor management and ergonomics principles	Wellbeing
Factors associated with post-traumatic stress disorder of nurses exposed to coronavirus disease 2019 in China [42]	Wang YX, Guo HT, Du XW, Song W, Lu C, Hao WN.	Cross sectional/questionnaires	202	This study aims to analyze the factors potentially involved in the post-traumatic stress disorder level of healthcare workers such as nurses, who are most exposed to COVID-19 in China. Nurses exposed to COVID-19 with job satisfaction and positive coping had low PCL-C scores. Effective and sustainable psychological counseling should be directed particularly to the female nurses in order to reduce the risk of psychological impairment	Job task: healthcare workers Organizational factors: work related stress factors	Negative psychological outcome
Perceived infection transmission routes, infection control practices, psychosocial changes, and management of COVID-19 infected healthcare workers in a tertiary acute care hospital in Wuhan: a cross-sectional survey [66]	Jin YH, Huang Q, Wang YY.	Cross sectional/electronic questionnaires	105	This study aims to explore the perceived pathways of infection, influencing factors, psychosocial changes, and management procedures of COVID-19 infected healthcare workers. Moreover, 88.3% of staff experienced psychological stress or emotional changes during their isolation period, only 11.7% had almost no emotional changes.	Job task: healthcare workers	Negative psychological outcome, quarantine related stress
The impact of having inadequate safety equipment on mental health [67]	Simms A, Fear NT, Greenberg N.	Cross sectional/online survey	3401	The purpose of this study is to evaluate the impact of inadequate safety equipment on the mental health of service staff, in order to better understand the impact on those working under the same conditions in response to COVID-19. Results indicate that psychological health problems are highly correlated with safety equipment perception.	Organizational factors: safety equipment perception	Negative psychological outcome
Academic Emergency Medicine Physicians’ Anxiety Levels, Stressors and Potential Stress Mitigation Measures during the Acceleration Phase of the COVID-19 Pandemic [68]	Rodriguez RM, Medak AJ, Baumann BM, Lim S, Chinnock B, Frazier R, Cooper RJ.	Cross sectional/online survey	426	The purpose of this research is to evaluate anxiety and burnout levels, home life changes and stress relief measures of United States academic emergency medicine (EM) doctors (Sample n. 426) during the acceleration phase of the COVID-19 pandemic. Most physicians (90.8%) reported changing their behavior towards family and friends, especially by decreasing signs of affection (76.8%). The most cited measures to alleviate stress/anxiety were increasing personal protective equipment (PPE) availability, offering rapid COVID-19 testing at the physician discretion’s, providing clearer communication about COVID-19 protocol changes, and assuring that physicians can take family and self-care leave.	Job task: healthcare workers. Organizational factors: PPE availability, COVID-19 management protocols	Negative psychological outcome
Mental Health Outcomes Among Frontline and Second-Line Health Care Workers During the Coronavirus Disease 2019 (COVID-19) Pandemic in Italy [69]	Rossi R, Socci V, Pacitti F, Di Lorenzo G, Di Marco A, Siracusano A, Rossi A.	Cross sectional/online questionnaires	681	This cross-sectional study analyzes mental health outcomes among healthcare workers in Italy. A total of 681 respondents (49.38%) endorsed post-traumatic stress symptoms; 341 (24.73%) symptoms of depression; 273 (19.80%) symptoms of anxiety; 114 (8.27%), insomnia; and 302 (21.90%) high perceived stress.	Job task: healthcare workers	Negative psychological outcome
COVID-19 Epidemic Peer Support and Crisis Intervention Via Social Media [46]	Cheng P, Xia G, Pang P, Wu B, Jiang W,	Descriptive study	N.A.	This article describes a support project developed and implemented by a group of mental health professionals (45 members of multidisciplinary healthcare professionals), organized to offer psychological support from overseas to professionals and healthcare workers at the forefront of the COVID-19 outbreak in Wuhan, China. Preliminary anecdotal review suggests that many of those served found the intervention helpful.	Organizational factors: work related stress factors (job support)	Mental wellbeing
Unravelling potential severe psychiatric repercussions on healthcare professionals during the COVID-19 crisis [70]	Anmella G, Fico G, Roca A, Gómez-Ramiro M, Vázquez M, Murru A, Pacchiarotti I, Verdolini N, Vieta E.	Case study/medical records review	1	The authors of this study report the case of a worker, a general practitioner, without a relevant somatic or psychiatric history who had a “brief reactive psychosis” under stressful circumstances derived from COVID-19.	Job task: healthcare workers	Insurgence of a brief reactive psychosis due to Covid-19 exposition
Factors Contributing to Healthcare Professional Burnout During the COVID-19 Pandemic: A Rapid Turnaround Global Survey [71]	Morgantini LA, Naha U, Wang H, Francavilla S, Acar O, Flores JM, Crivellaro S, Moreira D, Abern M, Eklund M, Vigneswaran H, Weine SM.	Cross sectional/online survey	2707	The aim of this research is to understand the risk for burnout in healthcare staff. This is critical to supporting HCPs and maintaining the quality of healthcare during the pandemic. Sample of 2707 HCPs from 60 countries. Fifty-one percent of HCPs reported burnout. Burnout was associated with work impacting household activities, feeling pushed beyond training, exposure to COVID-19 patients, making life-saving decisions. Adequate personal protective equipment (PPE) was protective against burnout.	Job task: healthcare workers Organizational factors: work-family conflict, risk of exposure to COVID-19, PPE availability	Negative psychological outcome and burnout syndrome
Geographical distance to the epicenter of Covid-19 predicts the burnout of the working population: Ripple effect or typhoon eye effect? [72]	Zhang SX, Huang H, Wei F.	Cross sectional/online survey	308	This study underlines how the geographical distance of adults working at the Wuhan epidemic center predicts their burnout-emotional, physical, and mental exhaustion due to excessive and prolonged stress. Preliminary results of a survey of 308 working adults in 53 cities showed working adults’ distance to the epicenter of Wuhan had an inverted U-shaped relationship with their burnout.	Organizational factors: working distance	Psychological outcomes
COVID-19 Impact Among Spine Surgeons in Latin America [73]	Guiroy A, Gagliardi M, Coombes N, et al.	Cross sectional/online questionnaires	204	This study investigated how COVID-19 pandemic impacts work performance and mental health of surgeons in Latin America. Twenty-two percent (*n* = 45) of the surgeons referred a mental status compatible with a depression diagnosis, especially for younger surgeons.	Job task: healthcare workers	Psychological outcomes
The distress of Iranian adults during the Covid-19 pandemic—More distressed than the Chinese and with different predictors [74]	Jahanshahi AA, Dinani MM, Madavani AN, Li J, Zhang SX.	Cross sectional/online survey	1058	This study investigated factors associated with mental distress in a sample of 1058 participants. Results showed that Iranian adults who worked from home, at the office, or had not worked during and before Covid-19, all reported lower distress that those who suspended working. In comparison, in China, only individuals who went to workplace reported significantly lower distress than those who suspended working.	Organizational factors: work modality and job task	Psychological outcomes

N.A.= Not available.

## 4. Discussion

The present narrative review focuses on the workplace related factors able to influence mental and psychological issue in the COVID-19 pandemic scenario. Several occupational factors were found as relevant to exacerbate or moderate the impact of COVID-19 on mental health of workers. What emerged from this review is that intrinsic high risk professional, organizational factors such as work related stress and lack of job support, and higher risk populations such as migrant workers and healthcare workers on the frontline are more likely to develop mental issue in the pandemic scenario.

The present narrative review focused on the workplace related factors capable of influencing to influence mental and psychological issues in the COVID-19 pandemic scenario. Several occupational factors were found as relevant to exacerbate or moderate the impact of COVID-19 on mental health of workers. What emerged from this review is the importance of high-risk professional and organizational factors, such as work-related stress and lack of job support, and the presence of populations at greater risk for mental health problems such as migrant workers. First of all, some helping professions, as in the case of health care professionals, expose workers to develop mental concerns due to their intrinsic higher risk. Most of the analyzed papers focused on the job task of healthcare workers. Moreover, it is noteworthy that some organizational factors can decrease the onset of mental issues, acting as moderators. The most vulnerable categories of workers seems to be front-line workers and health care workers, migrants, and young adult workers. The reduction of working time, the enhancement of smart working, the promotion of safe protocols, and the training and improvement of job/leadership support seems to be related to better performance and well-being. Above all, safety security and protection equipment seems to be highly and positive related to workers well-being and performance (6/42).

### 4.1. Workplace Related Factors and Mental Health in COVID-19 Pandemic among Healthcare Workers

Studies of other epidemics (SARS, MERS, Ebola) have shown that not only the general public suffers from emotional distress, but also many health professionals and law enforcement agents have faced symptoms of PTSD, depression, anxiety, exhaustion, and burnout at the beginning, during and after the outbreak [75]. Healthcare workers in the case of COVID-19 are more at risk for negative psychological consequences being equally susceptible to transmission due to inadequate individual protection devices (PPE), exhaustion, frustration, burnout, desperation, isolation, discrimination, negative emotion of patients, and distance of families [32]. World public health concerns many factors including the role and responsibility of healthcare professionals, the impact of infections, the impact of economic activities on travel and trade restrictions and the fair care of public welfare and individual rights during pandemics.

To decrease the extent of the psychological consequences, some actions can be taken: avoid intense exposure to COVID-19 media coverage (a phenomenon widely spread on an international scale) and maintain a compassionate and positive lifestyle by providing support to others. To deal with the side effects of the pandemic, resilience training programs should be implemented for healthcare professionals, law enforcement and the general public: (a) balance between family life and work; (b) clear and rapid information on the disease and its consequences on psychological well-being; (c) education and preparation of societies for pandemics and epidemics in the future; and (d) validation and evaluation of the contribution of frontline healthcare personnel [76].

Results from previous research that analyzed the psychological outcomes of epidemics, such as the 2003 SARS epidemic, show that up to 10% of healthcare professionals had SARS-related symptoms of PTSD even three years later [77]. To compare the magnitude, the 2003 SARS epidemic caused 774 victims from November 2002 to July 2003 with 8098 afflicted worldwide [78]. The COVID-19 pandemic caused around 83,947 deaths and infected 1,384,930 individuals in the United States alone from February 2020 to 15 May, 2020 [79]. This comparison highlights the profound impact that the COVID-19 pandemic could have on the psychological health of the entire healthcare sector. In details, eight specific sources of healthcare personnel anxiety related to the COVID-19 epidemic were argued, including (1) availability of appropriate personal protective equipment; (2) exposure to COVID-19 at work and bringing the infection home to family; (3) lack of access to testing if physicians develop COVID-19 symptoms and associated fear of propagating the infection at work; (4) uncertainty that physicians’ organization will take care of physicians personal needs if they become infected; (5) access to childcare during increased work hours and school closures; (6) lack of support for other personal and family needs as work demands increase; (7) being able to provide competent medical care if deployed to a new area; and (8) lack of access to up-to-date information and communication [80]. These sources of stress and anxiety do not fall within usual workplace scenario, leading to both burnout, and PTSD. In this way, the healthcare system and patient safety could be adversely affected by the worsening of systemic stressors [81].

### 4.2. Vulnerable Workers

This review highlighted the importance to properly address the risk in some vulnerable working populations such as migrant workers and frontline workers at higher risk of contagion. The professional community of internal migrant workers is vulnerable and prone to the onset of psychological effects due to a double impact: the COVID-19 crisis and the adverse employment environment [48]. Several factors interact with each other and predispose migrant workers to psychological distress and peri-traumatic symptoms. Possible stressors include susceptibility to new viral infections and the possibility of acting as vectors, pre-existing physical problems, such as professional pneumoconiosis, tuberculosis, HIV infections, pre-existing psychological morbidity, psychosocial factors, such as the absence of family support during the crisis, difficulty following personal safety regulations, isolation, and inability to receive psychiatric support promptly. This professional group appears to be further vulnerable to psychological distress due to factors such as financial constraints related to job loss and the absence or suspension of workplace safety and the basic laws related to occupational risks [48]. A recent study on SARS-CoV-2 compared, in a sample of 2299 respondents (mostly from the Chinese province of Fujian), the levels of fear, anxiety, and depression of social and health workers with those of administrative and managerial workers [82]. The results showed a significant imbalance towards the health figures who are most affected on a psychological level. In fact, the staff who worked in the high-risk wards (direct and prolonged contact with patients with SARS-CoV-2) showed a level of fear (*p* = 0.024), anxiety (*p* = 0.005) and depression (*p* = 0.007) significantly greater than non-clinical personnel and obviously greater anxiety (*p* = 0.026) than low-risk medical personnel. Despite this, stress levels should not be underestimated in any job category.

### 4.3. Organizational Factors and Target for Intervention

A socioscopic survey (with a valid sample of 673 subjects) administered to workers returning to their duties after the protracted lockdown, showed that 10.8% of respondents are facing a post-traumatic stress disorder, while they reported a low prevalence of anxiety (3.8%), depression (3.7%), stress (1.5%) and insomnia (2.3%) [55]. The World Health Organization (WHO) reiterates the need for those suffering from a mental disorder to have access to work, defining Psychosocial Rehabilitation as “a process that must facilitate individuals who have a damage or a disability due to a mental disorder, to develop all the opportunities to achieve the optimal level of independent functioning in the community”. According to the WHO, “psychosocial rehabilitation implies both an improvement of individual skills and the introduction of environmental changes, in order to create the conditions for the best possible quality of life”. However, the simple return to work represents only a first short step while a pivotal role will be played by the organization and the company. The survey by Tan and colleagues [59] showed that 95% of the respondent sample was less stressed and troubled if returning to a ventilated, sanitized, and prevention-conscious workplace. According to the results of Tan and colleagues, the factors associated with the severity of psychiatric symptoms in the workforce are marital status, the presence of physical symptoms, poor physical health, and the visualization of the return to work as a health hazard (*p* < 0.05). Consequently, a company that pays attention to the health of its operators will be able to experience a more fluid and simple return [59].

Most of the relevant scientific literature considered in our review has brought greater attention to the negative psychological and medical implications of the current pandemic [52,53,59]. In a smaller number of studies, possible solutions and management strategies applicable in the workplace were also considered. Furthermore, it seems that workplace research has exceeded in analyzing medical and nursing staff rather than companies and organizations broadly. However, the qualitative analysis of this review highlighted some useful exploitable strategies and methodologies in this pandemic. First of all, workplace emergency measures and safety equipment in response to COVID-19 have a positive relation with mental health and work performance of employees [47,67,71]. In addition, Dennerlein and colleagues (2020) highlighted how the Total Worker Health (TWH) approach, which includes human and psychological factors and ergonomic principles, supports workers’ safety, health, and psychological well-being during the COVID-19 pandemic [65].

To decrease the extent of the psychological consequences some actions can be taken: avoid intense exposure to COVID-19 media coverage (a phenomenon widely spread on an international scale) and maintain a compassionate and positive lifestyle by providing support to others. To deal with the side effects of the pandemic, resilience training programs should be implemented for healthcare professionals, law enforcement and the general public: (a) balance between family life and work; (b) clear and rapid information on the disease and its consequences on psychological well-being; (c) education and preparation of societies for pandemics and epidemics in the future; and (d) validation and evaluation of the contribution of frontline healthcare personnel [76].

This review has several limitations: studies sometimes do not fully specify the prevention and organization measures adopted in the workplace during the pandemic, so that it is hard to analyze the precise correlation between organizational measures and level of psychological problems. The studies analyzed come from countries with different levels of wealth, healthcare assistance and a different culture, so that the response to stress and crisis can be very different. Moreover, questionnaires and survey used to test the selected population can be very different from each other, even when investigating the same aspects. The selection of the population of each study considered may hide some bias as well as not being fully representative of the whole working population (for example: voluntary questionnaires administered online). Finally, psychological issues experienced by workers during the first state of emergency are subjected to change over time so that some future considerations about workplace organization in the future are difficult to establish.

Despite such limitations, this study has several points of strength. It attempts to connect work-related measures to the mental states of workers and to give some evidence on how organizational and work-related factors can contribute to maintenance or affect psychological well-being. Living and working in the era of COVID-19 is a challenge and supporting stakeholders in organizing the work environment and the safety protocols is a first step to get back to normality. The study identifies and tries to make a risk classification among workers, giving priorities in the interventions to come. Finally, it states out some correlation between work, social environment, and severe psychological diseases, pointing out relevant issues to attend in the field of Public Health. Further researches are needed to clearly understand all of these aspects.

## 5. Conclusions

Organizational and employment aspects have a considerable impact on psychological health, especially in the context of a global pandemic. The workplace therefore represents an important target towards which efforts should be directed to manage mental health issues related to the COVID-19 pandemic. Mental issues related to the health emergency, such as anxiety, depression, PTSD, suicidal ideas, sleep disorders, and drugs and alcohol addiction are more likely to affect healthcare workers, especially those on the frontline, migrant workers and workers in contact with the public, like the law enforcement. These issues are variously related to the high level of job strain, the fear of being infected and being a vector of the disease towards the family, the discrimination and stigma that may arise. Moreover, job insecurity, adverse employment environment, long periods of quarantine and isolation, work rights exploitations, and uncertainty of the future worsen the psychological condition, especially in younger people and in those with a higher educational background.

For these reasons, the public health response must address the issue of this so-called psychological pandemic, including support for psychological health, especially for higher risk populations and for those with pre-existing psychological disorders who are particularly vulnerable to pandemic stress.

Possible actions to mitigate the impact of the pandemic on the mental health of workers are the improvement of the infrastructures of workplace, the adoption of correct and shared anti-contagion measures, including regular PPE supply, the implementation of resilience training programs especially for workers with leadership roles. Monitoring mental health in different populations (onset and persistence of symptoms), understanding the different needs, and planning specific actions are also fundamental public health interventions.

In this scenario, promoting the development of reliable preventive approaches is essential. For example, the use of coaching psychology can be considered a valid strategy to lower burnout levels and create a safe environment in which individuals can feel free to discuss their professional development and understand how to improve their resources to overcome obstacles, such as the new challenges caused by the COVD-19 pandemic.
